# Adjuvant IL-7 potentiates adoptive T cell therapy by amplifying and sustaining polyfunctional antitumor CD4+ T cells

**DOI:** 10.1038/s41598-017-12488-z

**Published:** 2017-09-22

**Authors:** Zhi-Chun Ding, Tsadik Habtetsion, Yang Cao, Tao Li, Chufeng Liu, Michal Kuczma, Tingting Chen, Zhonglin Hao, Locke Bryan, David H. Munn, Gang Zhou

**Affiliations:** 1 Georgia Cancer Center, Medical College of Georgia, Augusta University, Augusta, Georgia USA; 2 Department of Orthodontics, Guangdong Provincial Key Laboratory of Stomatology, Hospital of Stomatology, Sun Yat-sen University, 56 Lingyuanxi Road, Guangzhou, PR China; 3 Department of Oncology Surgery, General Hospital of Ningxia Medical University, 804 Shengli Road, Yinchuan, PR China; 4 Department of Orthodontics, Guangdong Provincial Stomatological Hospital, Southern Medical University, S366 Jiangnan Boulevard, Guangzhou, PR China

## Abstract

Increased availability of homeostatic cytokines is considered a major mechanism by which lymphodepletion enhances the efficacy of adoptive T cell therapy (ACT). IL-7 is one such cytokine capable of augmenting the function of tumor-reactive CD8+ T cells. However, whether host-derived IL-7 plays a role in driving the proper function of CD4+ T cells in an ACT setting remains unclear. Here we report that lymphodepleting chemotherapy by cyclophosphamide (CTX) does not lead to increased availability of the endogenous IL-7 in mice. Despite of a paucity of IL-7 in the immune milieu, CTX preconditioning allowed adoptively transferred naïve tumor-specific CD4+ T cells to undergo effector differentiation and regain IL-7Rα expression, giving rise to IL-7-responsive polyfunctional CD4+ effector cells. Correspondingly, supplementation of exogenous recombinant IL-7 markedly amplified and sustained polyfunctional CD4+ effector cells, resulting in improved therapeutic outcome in a mouse lymphoma model. We further demonstrated that the immune-enhancing effects of IL-7 were also applicable to donor CD4+ T cells pre-activated under Th1 polarizing condition. These findings suggest caution in relying on the endogenous IL-7 to enhance donor T cell expansion and persistence after lymphodepleting chemotherapy, and highlight the usefulness of recombinant IL-7 as an adjuvant for adoptive immunotherapy.

## Introduction

IL-7 is a hematopoietic growth factor involved in regulating multiple aspects of T cell biology including survival, homeostasis, metabolism and memory^1,2^. Under the steady state, a limited amount of IL-7 is produced by non-hematopoietic cells and consumed by various types of cells that express a heterodimeric receptor consisting of interleukin-7 receptor α (IL-7Rα) and common-γ chain receptor^3^. Lymphopenic conditions in human and mice are associated with increased levels of IL-7 in the circulation likely due to decreased consumption. Rag1−/− and IL-7Rα−/− mice have elevated serum IL-7 compared to wild-type mice^4^. In humans, increased levels of IL-7 are observed in individuals with lymphopenia due to genetic disorders such as severe combined immune deficiency (SCID)^5^. Higher IL-7 levels have also been detected in patients who received high dose chemotherapy regimens prior to bone marrow transplantation or hematopoietic stem-cell transplantation^5–7^. In the setting of adoptive T-cell therapy (ACT) for cancer, it has been shown that augmentation of ACT efficacy by total body irradiation (TBI) relies on adoptively transferred CD8+ T cells to respond to host-derived IL-7^8,9^. Likewise, IL-7 released after lymphodepleting cyclophosphamide (CTX) chemotherapy has been implicated in enhancing the homing and proliferation of the donor T cells^10^.

Mounting evidence indicates that CD4+ T cells can mediate tumor destruction through multiple mechanisms. CD4+ T cells can act as effector cells to execute direct tumor lysis through granzyme B^11,12^. CD4+ T cells can potentiate the activation of other tumor-reactive immune cells via CD40L expression and by release of inflammatory cytokines including IFNγ, IL-2 and TNFα^13–20^. In addition, CD4+ T cells can remodel the tumor microenvironment, creating an immune milieu that is hostile to tumor growth^21,22^. CD4+ T cell-based ACT has advanced into the clinical arena and shown impressive therapeutic potential in several clinical studies^23,24^. We and others previously reported that host preconditioning with CTX or TBI allows adoptively transferred tumor-specific CD4+ T cells to differentiate into polyfunctional effector cells characterized by their ability to concomitantly express multiple effector molecules including CD40L, IFNγ, IL-2, TNFα and granzyme B^11,25–27^. In this study, we seek to investigate if induction of polyfunctional CD4+ T cells relies on increased IL-7 availability resulted from lymphodepleting preparative chemotherapy. We report the surprising finding that CTX-based lymphodepleting chemotherapy does not lead to a measurable increase in IL-7 availability. In addition, we show that supplementation of exogenous IL-7 promotes the expansion and maintenance of *in vivo*-differentiated polyfunctional CD4+ effector cells or *in vitro*-generated Th1 cells, supporting the use of IL-7 as an adjuvant for adoptive immunotherapy.

## Results

### Lymphodepleting preparative chemotherapy allows adoptively transferred tumor-specific CD4+ T cells to differentiate into polyfunctional effector cells and regain IL-7Rα expression

**Figure 1:**
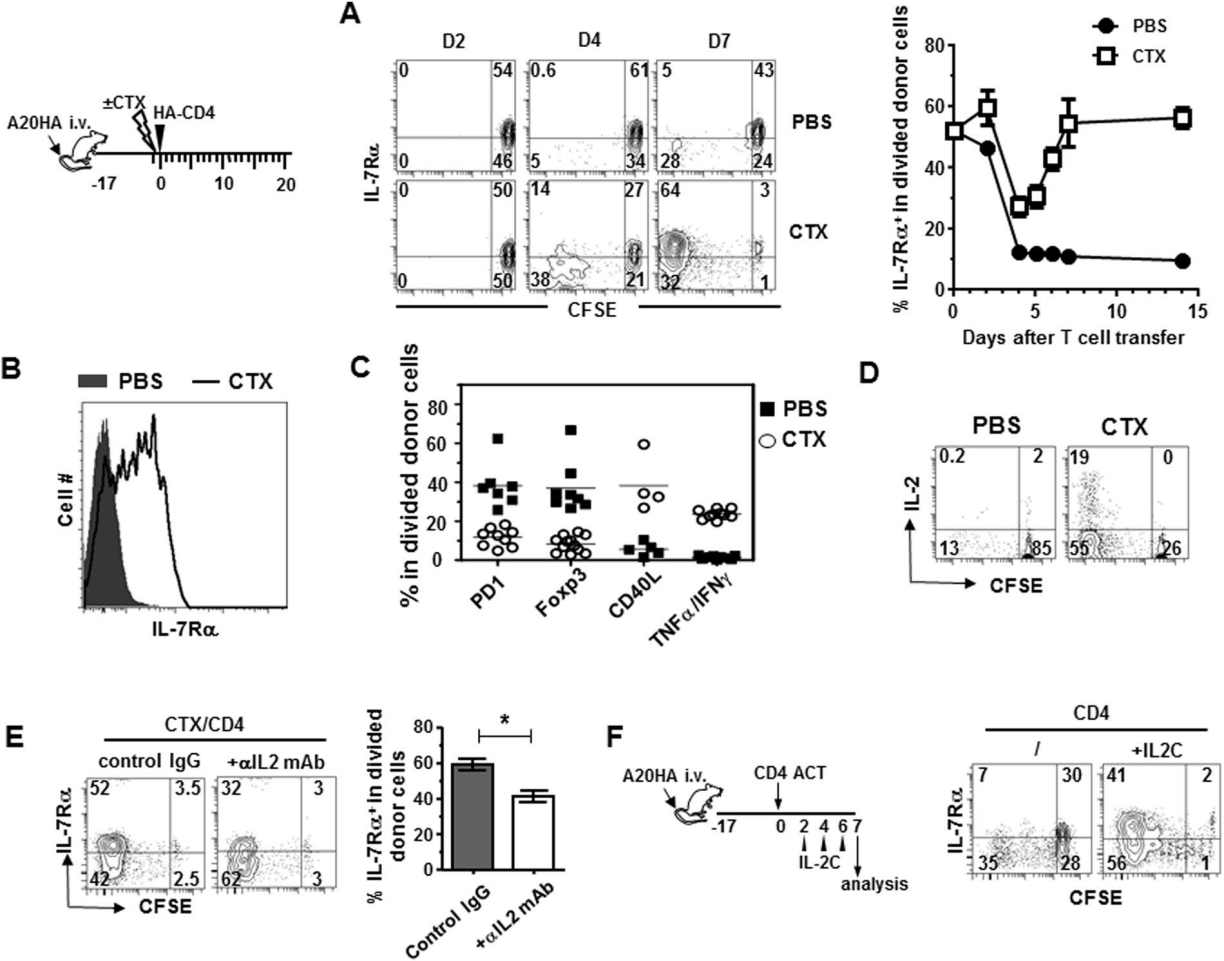
Host pre-conditioning with CTX allows adoptively transferred tumor-specific CD4+ T cells to differentiate into polyfunctional effector cells and regain IL-7Rα expression. (**A**) Kinetics of IL-7Rα expression on donor CD4+ T cells following adoptive transfer. Following the timeline depicted in the schema, mice with established systemic A20HA tumors were divided into two groups. One group of mice were pre-conditioned with CTX while the other group received PBS. All mice received adoptive transfer of HA-specific CD4+ T cells the next day. At the indicated time points, tail blood samples were collected and analyzed for IL-7Rα expression on donor CD4+ T cells by FACS. IL-7Rα expression profiles relative to cell division in transferred CD4+ T cells are shown in representative dot plots. The numbers indicate the percentage of cells in the corresponding quadrant. Results are summarized in graph at right. Data for day 0 and day3 are gated on total donor T cells because cells barely divided at these two time points. (**B**) Differential expression of IL-7Rα in donor CD4+ T cells in mice with or without CTX pre-conditioning. 7 days after T-cell transfer, spleen cells were isolated to examine IL-7Rα expression on donor CD4+ T cells. Donor T cells were also evaluated for expression levels of PD1, Foxp3, CD40L, IFNγ and TNFα. The phenotypes of the divided donor T cells in PBS or CTX-conditioned mice are summarized in (**C**). (**D**) Donor CD4+ T cells transferred into CTX-conditioned mice but not unconditioned mice are capable of producing IL-2. IL-2 expression profiles relative to cell division in transferred CD4+ T cells are revealed by intracellular staining (ICS). (**E**) IL-2 neutralization inhibits IL-7Rα re-expression in donor CD4+ T cells in CTX-conditioned mice. Tumor-bearing mice were treated with CTX followed by CD4+ T cell transfer the next day. Some mice were injected with IL-2 neutralizing mAbs every other day. 7 days after T cell transfer, IL-7Rα expression on donor CD4+ T cells were analyzed by FACS. Results summarized in bar graph are shown as mean ± SD of 3 mice each group. (**F**) Exogenous IL-2 partially restores IL-7Rα expression in donor CD4+ T cells transferred into unconditioned mice. Following the timeline depicted in the schema, A20HA tumor-bearing mice received adoptive transfer of HA-specific CD4+ T cells. A cohort of mice further received IL-2/αIL-2 complex (IL-2C) every other day. IL-7Rα expressions on donor CD4+ T cells were analyzed 7 days after T cell transfer.

Bracci *et al*. reported that CTX treatment resulted in release of a multitude of cytokines and chemokines, i.e. a “cytokine storm”, among which IL-7 contributed to donor T cell homing and proliferation^10^. Currently, it is unclear how IL-7 shapes the fate of CD4+ T cells, which have the option to become either helper/effector cells or regulatory T cells (Tregs). Our previous work showed that host preconditioning with CTX allows adoptively transferred tumor-specific CD4+ T cells to differentiate into polyfunctional effector cells^25^. Using this model, we first set out to investigate the IL-7 responsiveness of the donor CD4+ T cells by examining their IL-7Rα expression during effector differentiation. As shown in Fig. 1 schema, mice with established systemic HA-expressing A20 lymphoma (A20HA) were either untreated or treated with CTX followed by adoptive transfer of naïve HA-specific CD4+ T cells. Figure 1A shows that most donor CD4+ T cells detected in peripheral blood remained undivided 2 days after transfer. By day 4, most donor CD4+ T cells in CTX-treated hosts have divided with a majority of the divided cells having reduced IL-7Rα expression compared to undivided donor cells. The fraction of divided CD4+ T cells increased further by day 7 and the majority of these divided cells regained IL-7Rα expression to the level comparable to that in naïve CD4+ T cells. In sharp contrast, donor CD4+ T cells downregulated IL-7Rα upon antigen recognition in unconditioned tumor-bearing mice and IL-7Rα remained suppressed in these mice. A cohort of mice were sacrificed on day 7 to analyze the phenotype and function of the donor T cells in the spleen. Consistent with the IL-7Rα expression pattern of donor T cells in blood, the divided donor CD4+ T cells from the spleens of CTX-treated mice were mostly IL-7Rα^high^, whereas their counterparts in the spleens of unconditioned mice were IL-7Rα^low^ (Fig. 1B), suggesting that the changes in IL-7 responsiveness in donor CD4 T cells are systemic and pertinent to the environment conditioned by chemotherapy. We further showed that donor CD4+ T cells in CTX-treated mice acquired a polyfunctional effector phenotype, as reflected by low levels of PD1 and Foxp3, increased expression of CD40L, and the ability to concomitantly produce IL-2, TNFα and IFNγ (Fig. 1C). In contrast, donor CD4 T cells from unconditioned mice exhibited a tolerized phenotype, characterized by high expression of PD1 and Foxp3, low level of CD40L, and inability to produce proinflammatory cytokines. The results indicate that CTX preconditioning is a prerequisite for donor CD4+ T cells to acquire IL-7 responsiveness, which correlates with the functional status of tumor-reactive CD4+ T cells.

We sought to determine the factor(s) that controls IL-7Rα re-expression in activated CD4+ T cells. Studies from two groups present evidence that autocrine IL-2 plays an important role in regulating IL-7Rα re-expression in primed CD4+ T cells. Dooms *et al*. reported that IL-2−/− or CD25−/− (IL-2Rα−/−) CD4+ T cells failed to re-express IL-7Rα after antigenic stimulation *in vitro* and primed IL-2−/− or CD25−/− CD4+ T cells developed poorly into memory cells *in vivo* ^28^. McKinstry *et al*. reported that in an influenza infection mouse model, IL-2 was essential for IL-7Rα upregulation on CD4 effector cells during the priming phase, enabling IL-7-dependent effector to memory transition at the late stage^29^. In line with these studies, we found that IL-7Rα re-expression on donor CD4 T cells in CTX-treated mice correlated with the ability of donor T cells to produce IL-2, whereas IL-7Rα remained suppressed in IL-2-silenced donor CD4 T cells in unconditioned mice (Fig. 1D). We tested whether the expression pattern of IL-7Rα in donor CD4 T cells can be altered by manipulating the presence or absence of IL-2 in mice. To this end, we injected IL-2 neutralizing monoclonal antibodies to tumor-bearing mice that received adoptive transfer of tumor-specific CD4+ T cells following CTX preconditioning. Figure 1E shows that neutralization of IL-2 led to reduced IL-7Rα expression on donor CD4+ T cells that have encountered tumor Ag and divided. Next, we administered IL-2/αIL-2 complex (IL-2C) to tumor-bearing mice that received tumor-specific CD4+ T cells without chemotherapy preconditioning (Fig. 1F schema). Figure 1F shows that supplementing exogenous IL-2 markedly restored IL-7Rα expression on donor CD4+ T cells that would otherwise remain suppressed for IL-7Rα. Our data suggest that IL-2 plays an important role in regulating the IL-7 responsiveness of donor CD4+ T cells in the post-chemotherapy period.

### Measurements of host-derived endogenous IL-7 in post-chemotherapy milieu

**Figure 2:**
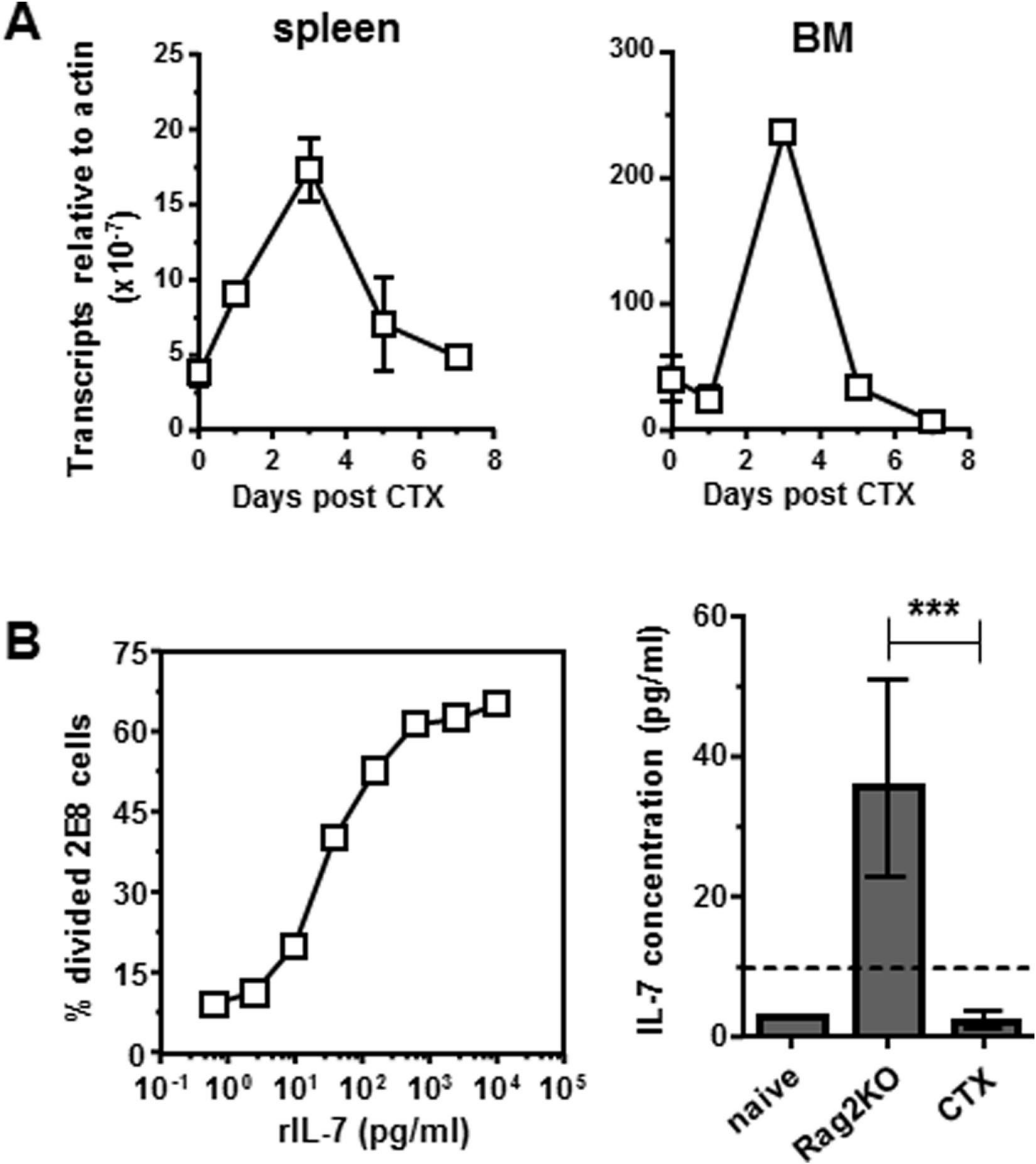
Quantification of the endogenous IL-7 in mice after CTX treatment. (**A**) IL-7 RNA levels in mouse spleen and bone marrow (BM) after CTX treatment. At the indicated time points after CTX treatment, spleen and BM samples were harvested, and total RNA was extracted. IL-7 transcripts in spleen and BM was quantified by real-time RT-PCR. Data represent the relative amount of IL-7 mRNA normalized to β-actin shown as mean ± SD of triplicates of each sample. Data shown are results of one out three independent experiments with similar results. (**B**) Quantification of IL-7 levels in mouse serum using 2E8 cell-based bioassay. Serum samples were collected from Rag2KO, untreated naïve mice, and CTX-treated mice. CFSE-labeled IL-7-responsive 2E8 cells were seeded in a 96-well plate in the presence of serum samples or serially diluted rmIL-7 as the standards. After 3 days in culture, cell proliferation was evaluated by CFSE dilution using flow cytometry. The graph on the left shows the standard curve, which is generated by plotting the percent of divided 2E8 cells against the concentration of rmIL-7. The bar graph on the right shows IL-7 concentrations in serum samples calculated based on the regression equation derived from the standard curve. The dash line indicates the concentration of rmIL-7 needed in culture in order to drive 2E8 cell proliferation that is significantly above the background level. Data shown are pooled from 2 independent experiments with at least lest 3 mice in each group. ***P < 0.001.

As a surge of IL-7 was expected after lymphodepleting chemotherapy, we decided to determine the level of IL-7 in the post-chemotherapy immune milieu. We first evaluated IL-7 transcripts in the spleens and bone marrows (BM) harvested from mice at different time points after CTX treatment. Quantitative RT-PCR assays indicate that after CTX treatment, IL-7 transcripts were significantly induced in both spleen and BM, reaching the peak on day 3, then returning to baseline levels by day 7 (Fig. 2A) exhibiting an expression pattern similar to that reported by Bracci *et al*.^10^. For IL-7 protein, we attempted but failed to detect IL-7 in serum from CTX-treated mice using a commercial ELISA kit which had a detection limit of 30 pg/ml (data not shown). Guimond *et al*. described detection of IL-7 as low as 10 pg/ml using a bioassay based on the proliferation of an IL-7-dependent cell line 2E8^4^. Adopting this approach, we established a standard curve correlating 2E8 proliferation to serially diluted recombinant IL-7 concentration (Fig. 2B, left graph). Using this method for IL-7 measurement, none of the serum samples collected at different time points after CTX treatment contained IL-7 above the detection limit (data not shown). The calculated average IL-7 concentration in serums collected on day 3, the time when mice had the lowest lymphocyte counts^30,31^, was 3.2 pg/ml (±1.2 pg/ml) which was indistinguishable from the IL-7 level in untreated WT mice (Fig. 2B, right graph). Of note, Rag2KO mice had elevated level of serum IL-7 (36 ± 25 pg/ml) as previously reported^4^, validating the sensitivity and reliability of the IL-7 measurement method.

### Lymphodepleting chemotherapy does not lead to increased availability of IL-7 in mice

**Figure 3:**
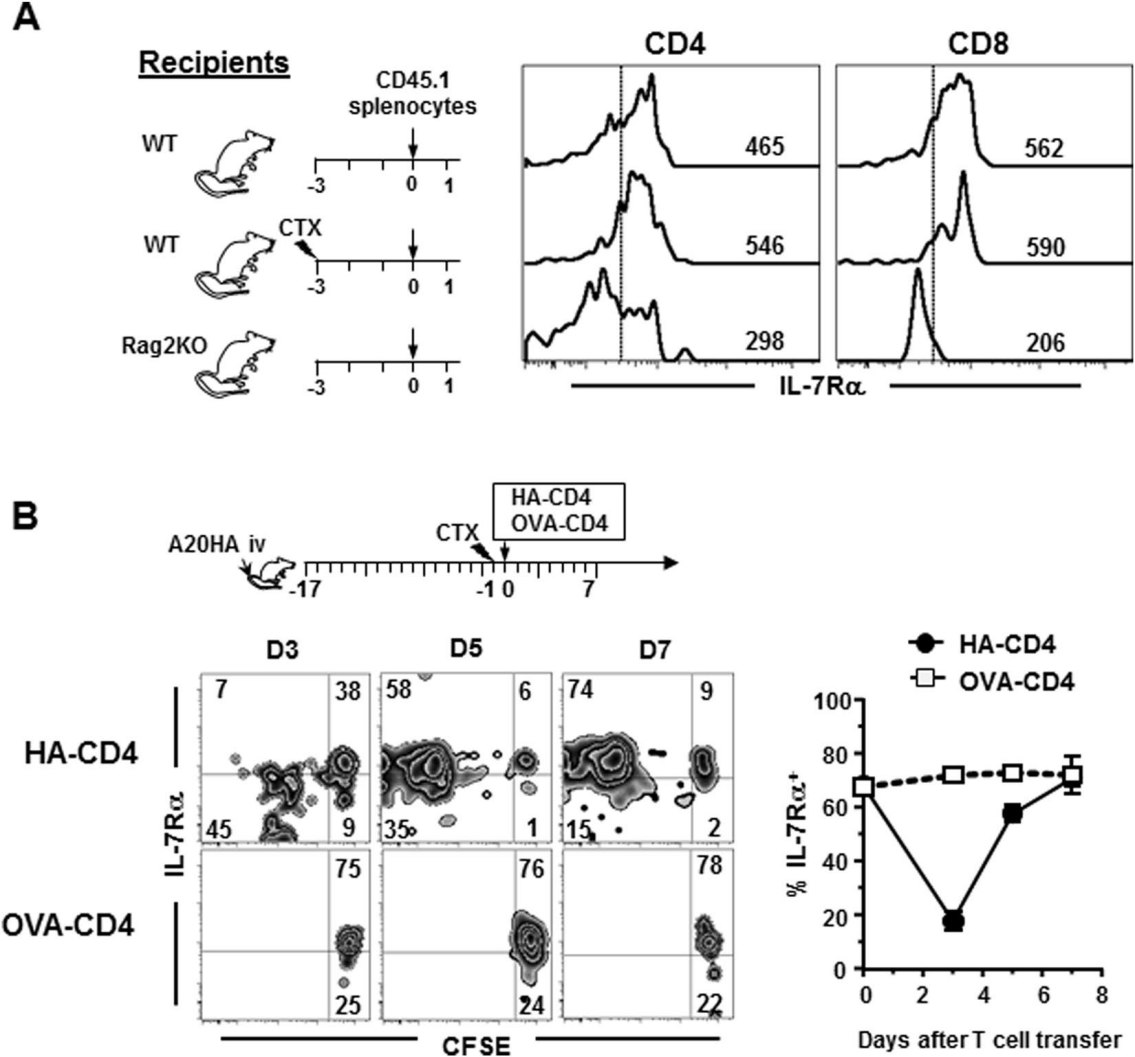
Host preconditioning by CTX does not lead to increased availability of IL-7 in mice. (**A**) The IL-7 levels in Rag2KO mice but not CTX-treated mice lead to downregulation of IL-7Rα in adoptively transferred naïve T cells. Following the timeline depicted in the schema, splenocytes from CD45.1 mice were transferred into untreated mice (WT), CTX-treated mice, and Rag2KO mice. 16 hours later, peripheral blood and spleen samples were collected and examined for expression of IL-7Rα in transferred CD4+ and CD8+ T cells by flow cytometry. Numbers in representative histograms indicate the values of mean florescence intensity (MFI) of IL-7Rα. (**B**) The dynamic changes in IL-7Rα expression in donor T cells are driven by antigen encounter in CTX-conditioned mice. As shown in the schema, A20HA tumor-bearing mice were treated with CTX followed by co-transfer of HA-specific and OVA-specific CD4+ T cells. Tail blood was collected at the indicated time points and examined for IL-7Rα expression by FACS. IL-7Rα expression profiles relative to cell division in HA-specific and OVA-specific CD4+ T cells are shown in representative dot plots. Results from two independent experiments are summarized in graph at right.

Our results indicated an absence of IL-7 surge in the post-chemotherapy milieu which is in contradiction to the perception that IL-7 is enriched in lymphopenic hosts. We thus further evaluated the level of IL-7 and its physiological significance in mice treated with CTX. It has been shown that the level of IL-7 in Rag2KO mice is sufficient to cause IL-7Rα downregulation in adoptively transferred naïve T cells^4^. We wanted to test if chemotherapy induces an increase in IL-7 to levels sufficient to cause IL-7Rα downregulation in transferred naïve T cells. Splenocytes from CD45.1 congenic mice were transferred into untreated naive mice, CTX-treated mice, and Rag2KO mice (Fig. 3C schema). Cell transfer was performed 3 days after CTX treatment to coincide with the host lymphocyte nadir, thereby reducing a potential competing population and maximally exposing the donor T cells to the available endogenous IL-7. IL-7Rα expression on donor T cells was analyzed by FACS 16 hours after cell transfer. As expected, donor CD4+ and CD8+ T cells had reduced IL-7Rα expression when transferred into Rag2KO but not untreated naive recipients (Fig. 3A), validating the IL-7 detection sensitivity of the methodology. Notably, donor T cells in CTX-treated mice did not downregulate IL-7Rα expression, indicating that host-derived IL-7 in CTX-treated mice does not reach the level of IL-7 in Rag2KO mice.

The preceding experiment was performed in tumor-free mice where the donor T cells did not encounter their cognate antigens. It is conceivable that in the setting of ACT, tumor-specific CD4+ T cells would receive antigenic stimulation while being exposed to increased levels of endogenous IL-7. Given that IL-7Rα expression is regulated by both TCR and IL-7 signaling^32–34^, we went on to delineate the impact of TCR and/or IL-7 signaling on the IL-7 responsiveness of tumor-specific CD4+ T cells as well as bystander T cells. We co-transferred HA-specific CD4+ T cells (6.5CD4) and irrelevant OVA-specific CD4+ T cells (DO11.10CD4) to CTX-conditioned A20HA tumor-bearing mice (Fig. 3B schema). Under this condition, both donor populations were exposed to the same immune milieu while only HA-specific CD4+ T cells can undergo antigen-driven proliferation. Figure 3B shows that IL-7Rα in HA-specific CD4+ T cells had an initial downregulation followed by re-expression, whereas OVA-specific CD4 T cells remained undivided and maintained high levels of IL-7Rα. The data suggest that the endogenous IL-7 during the course of ACT never reached a level sufficient to trigger IL-7Rα downregulation in bystander naïve T cells (OVA-specific CD4). In addition, the drop/rebound IL-7Rα expression pattern in tumor-specific CD4+ T cells is likely driven by TCR signaling alone. Indeed, it has been shown by others that upon antigenic stimulation *in vitro* or *in vivo*, CD4+ T cells initially lose IL-7Rα but later regain its expression^28,29^.

### Adjuvant IL-7 potentiates the efficacy of adoptive CD4+ T-cell immunotherapy by augmenting the expansion and persistence of polyfunctional CD4+ effector cells

**Figure 4:**
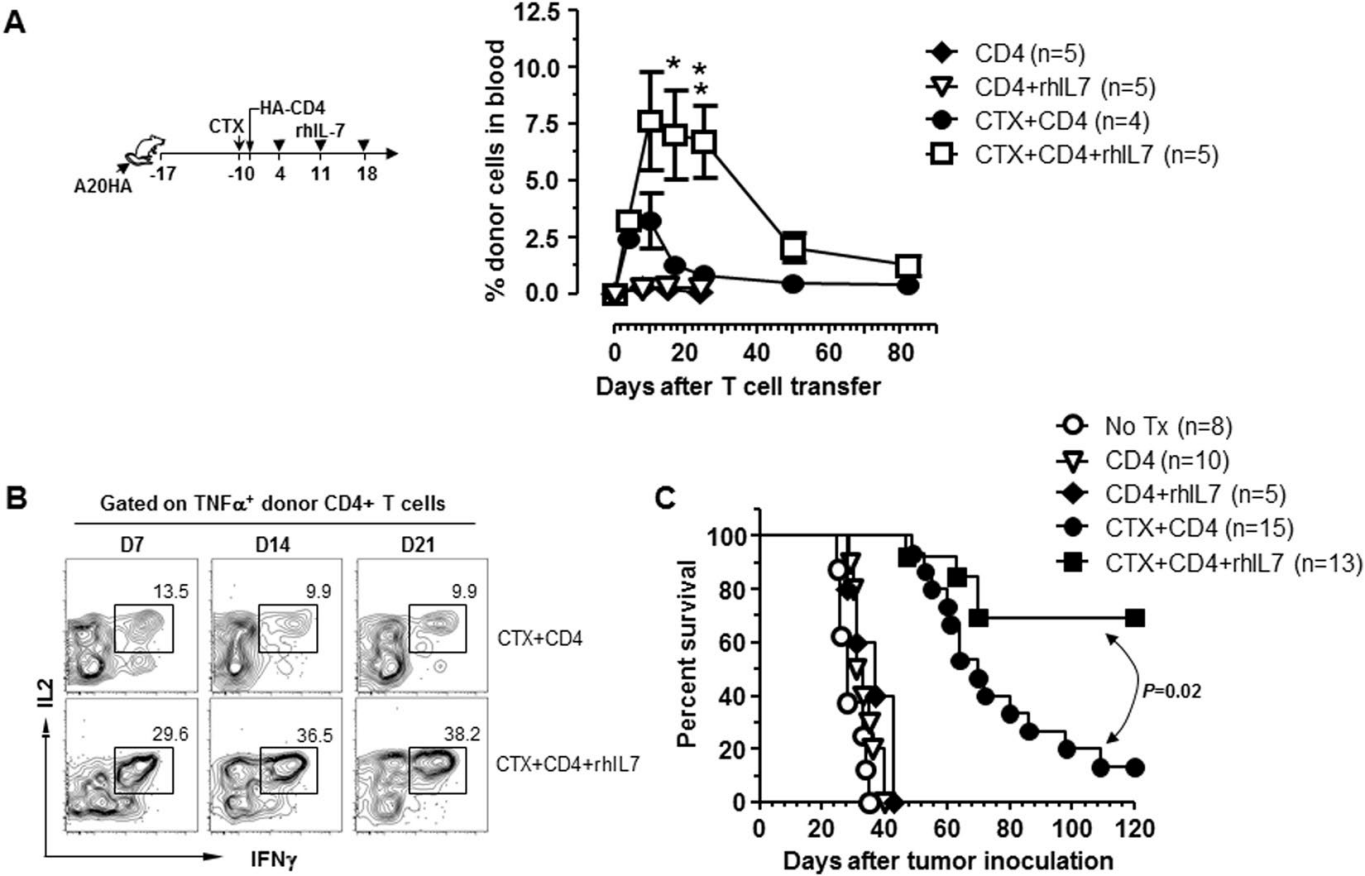
Adjuvant IL-7 potentiates the efficacy of adoptive CD4+ T-cell transfer by augmenting the expansion and persistence of polyfunctional CD4+ effector cells. Following the timeline depicted in the schema, mice with established systemic A20HA tumors were randomly grouped and received either no treatment (no Tx) or HA-specific CD4+ T-cell transfer (CD4) under one of the following conditions: CD4+ T-cell transfer only (CD4), CD4+ T-cell transfer and rhIL-7 administration (CD4+ rhIL-7), CTX pre-conditioning and CD4+ T-cell transfer (CTX + CD4), CTX pre-conditioning and CD4+ T-cell transfer plus rhIL-7 administration (CTX + CD4+ rhIL-7). (**A**) Administration of rhIL-7 enhances the expansion and duration of the donor CD4+ T cells in CTX-conditioned mice. Tail blood collected at the indicated time points were analyzed for donor T cell frequencies by FACS. Results shown are representative of two independent experiments with 5 mice in each group. *P < 0.05. **P < 0.01. (**B**) rhIL-7 administration sustains the polyfunctionality of the donor CD4+ T cells. Tail blood was collected at the indicated time points and subjected to cytokine ICS analysis. Plots shown are gated on TNFα^+^ divided donor CD4+ T cells. The numbers represent the percentage of cells co-expressing TNFα, IL-2 and IFNγ. Results shown are representative of two independent experiments. (**C**) rhIL-7 administration potentiates the efficacy of CD4+ T-cell transfer in CTX-conditioned mice. The Kaplan-Meier plot depicts the overall survival of mice under each treatment condition. Results shown are pooled data from 3 independent experiments.

The above data implied that there was a relative paucity of host-derived IL-7 in the immune milieu following lymphodepleting chemotherapy. Nonetheless, chemotherapy conditioning created an immunogenic environment within which donor CD4+ T cells encountered antigenic stimulation, giving rise to polyfunctional CD4+ effector cells capable of responding to IL-7. Since the adjuvant effects of IL-7 in enhancing cancer vaccine-induced T-cell responses have recently been demonstrated^35,36^, we posit that the IL-7Rα^+^ polyfunctional CD4+ effector cells arising after ACT would be amenable to adjuvant IL-7. To test this hypothesis, we used our established CD4+ ACT model to examine whether administration of exogenous IL-7 can improve the therapeutic outcome by boosting the expansion of polyfunctional CD4+ T cells. As shown in Fig. 4 schema, some mice bearing late-stage systemic A20HA tumors were pre-conditioned with CTX, followed by adoptive transfer of HA-specific CD4+ T cells the next day. Recombinant human IL-7 (rhIL-7) was administered weekly to a cohort of mice for a total of 3 injections, starting 3 days after T cell transfer. Mouse tail blood was collected at different time points and assayed for the frequencies and polyfunctionality of the donor CD4+ T cells. Notably in these CTX-conditioned mice, rhIL-7 administration significantly enhanced the expansion and delayed the contraction of the donor CD4+ T cells (Fig. 4A, CTX + CD4+ rhIL-7 vs CTX + CD4). As shown in Fig. 4B, 10–15% of the transferred CD4+ T cells remained polyfunctional in mice that did not receive rhIL-7 administration; strikingly, rhIL-7 injection not only increased the frequency (30–40%) of polyfunctional T cells within the donor CD4+ population, but also maintained this high level of polyfunctionality for an extended period of time (at least 3 weeks). Using mouse survival as the functional readout, Fig. 4C shows that although CTX + CD4 ACT significantly prolonged the lifespan of tumor-bearing mice as we reported previously^25,26^, the majority of mice (87%) in this cohort had late relapse and died by day 120. In contrast, rhIL-7 administration following CTX + CD4 ACT largely reduced the occurrence of relapse and led to long-term survival in the majority of mice (70%). As controls, donor CD4+ T cells neither expanded nor responded to rhIL-7 after transferring into unconditioned tumor-bearing mice (Fig. 4A, CD4 and CD4+ rhIL-7). Moreover, these conditions did not alter the kinetics of mouse survival compared to untreated mice (Fig. 4C), indicating that the observed beneficial effects resulted from the synergy between CTX-conditioning, CD4 ACT, and administration of rhIL-7.

### CD4+ T cells activated under the Th1 polarizing condition respond to rhIL-7 after transferring into CTX-conditioned tumor-bearing hosts

**Figure 5:**
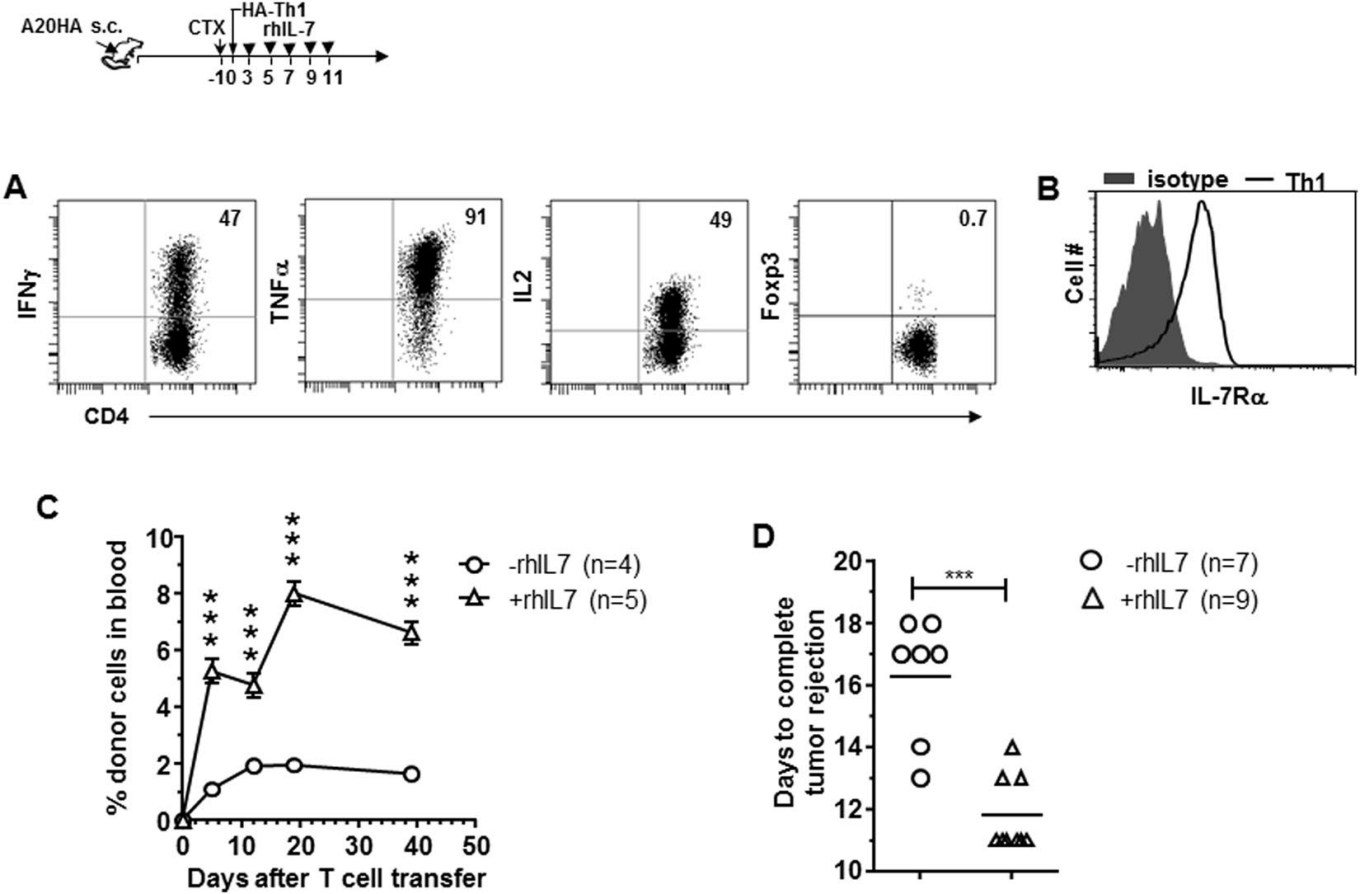
CD4+ T cells activated under the Th1 polarizing condition respond to rhIL-7 after transferring into CTX-conditioned tumor-bearing hosts. The schema outlines the timeline of experimental procedures. Balb/c mice were inoculated with A20HA tumors subcutaneously in one flank. When tumor sizes reach ~170 mm^2^, mice were treated with CTX (150 mg/kg) followed by adoptive transfer of *in vitro*-differentiated HA-specific Th1 CD4 T cells (1 × 10^6^ Th1 cells/mouse). A cohort of mice were then subcutaneously injected rhIL-7 (10 ug/injection) at the indicated time points. (**A**) Phenotypic characterization of *in vitro*-cultured HA-specific Th1 CD4 T cells. Cytokine profile and Foxp3 level of the cultured Th1 cells were evaluated by FACS. Numbers indicate the percentage of cells positive for the assayed marker. (**B**) IL-7Rα expression on *in vitro*-cultured HA-specific Th1 CD4+ T cells before adoptive transfer. (**C**) rhIL-7 administration enhances the expansion and maintenance of the infused Th1 cells. Tail blood samples collected at the indicated time points were analyzed for donor Th1 cell frequencies by FACS. (**D**) rhIL-7 administration leads to accelerated complete tumor rejection. The numbers of day needed to achieve complete tumor rejection are plotted. Results are pooled from 2 independent experiments. ***P < 0.001.

It should be noted that the above experiments used unstimulated naïve CD4+ T cells as donor cells for ACT. However, the donor T cells used in human ACT trials often receive stimulations during *ex vivo* expansion or genetic modification, and thus are mostly activated T cells at the time of infusion. To simulate this scenario, we stimulated tumor-specific CD4+ T cells under the Th1 polarizing condition and infused the cell products to CTX-conditioned tumor-bearing mice, with or without subsequent rhIL-7 administration (Fig. 5 schema). The donor T cells exhibited the expected Th1 phenotype, i.e. IFNγ^+^TNFα^+^IL2^+^Foxp3^−^ (Fig. 5A), and had regained IL-7Rα expression at the time of transfer (Fig. 5B). Figure 5C shows that rhIL-7 administration not only boosted the expansion of the infused Th1 cells but also maintained these cells at higher levels for a sustained period compared to the control group. In this tumor model, adoptive transfer of 1 × 10^6^ Th1 cells following CTX led to complete tumor rejection of large established A20HA tumors implanted in the flank oin mice. Notably, rhIL-7 administration significantly shortened the time needed to achieve complete tumor rejection compared to the control group (Fig. 5D, 11.7 ± 0.4 days vs. 16.3 ± 0.8 days). The data suggest that ACT using previously activated CD4+ T cells can also benefit from the adjuvant effect of rhIL-7.

## Discussion

ACT has proven to be an effective treatment strategy for certain types of cancer^37,38^. It has been well-established that lymphodepleting conditioning of the host prior to ACT promotes engraftment, expansion and persistence of the donor T cells^39^. TBI and CTX-based preparative chemotherapy are the two major means for host lymphodepleting conditioning. The benefits of host conditioning are, at least in part, attributable to exposure of donor T cells to increased levels of growth factors, as the result of removing the endogenous homeostatic “cytokine sinks” that normally contribute to peripheral tolerance^40^. Among the cytokines enriched in lymphopenic recipients, IL-7 is believed to play a critical role in enhancing the survival and function of the donor T cells^41,42^.

Given the long-held perception that lymphopenia in mice and humans is associated with increased IL-7 in circulation, our data showing no overt increase in IL-7 availability after lymphodepleting chemotherapy is somewhat surprising. It has been well-established that CTX-mediated host conditioning can potentiate ACT efficacy^43,44^. The idea that IL-7 plays a role in CTX’s immunopotentiating effect is supported by a study showing that IL-7 transcripts were elevated in mouse BM and spleen after CTX treatment, and IL-7 neutralization reduced the number of donor T cells in mice^10^. Intriguingly, prior studies did not provide direct measurement of the IL-7 levels in mice rendered lymphopenic by CTX. Moreover, an increase of IL-7 was not detected in a comprehensive protein expression profiling analysis performed on BM and plasma samples collected from CTX-treated mice^31^. In humans, although higher IL-7 levels were detected in patients who received high dose chemotherapy regimens prior to autologous hematopoietic stem-cell transplantation (ASCT), the changes in endogenous IL-7 levels in patients upon chemotherapeutic treatment before ASCT were rather low with an increase from around 2 pg/ml up to 10 pg/ml^7^. Here, our data indicate that the level of serum IL-7 in CTX-treated mice is less than that in Rag2KO mice (~30 pg/ml). Nevertheless, our results do not exclude the possibility of increased IL-7 synthesis since IL-7 transcripts were indeed increased after CTX (Fig. 2A), which is consistent with published studies^10^. However, the increases of IL-7, either due to increased synthesis and/or reduced consumption, did not lead to accumulation of IL-7 to a level that was detectable by our bioassays, nor did it cause IL-7Rα downregulation in surrounding T cells. It is also possible that the level of IL-7 is correlated with the degree of lymphopenia. The doses of CTX used in most mouse ACT models fall in the range of 80 mg/kg–200 mg/kg^44^, which are lymphodepleting but non-myeloablative. Increasing the CTX dose to 300 mg/kg resulted in only slight increase of serum IL-7 compared to 150 mg/kg CTX in some but not all mice (data not shown). It is conceivable that induction of significantly increased IL-7 in circulation may require high dose chemotherapies that lead to severe cytopenias^5–7^.

As a potent T-cell growth factor, IL-7 has been considered a promising immunotherapy drug with benefits in promoting immune reconstitution in patients with prior lymphodepleting chemotherapy^41,45^. rhIL-7 administered to patients exhibited a favorable safety profile, preferentially expanded circulating naive CD4+ and CD8+ T cells but not Treg cells, and led to increased TCR repertoire diversity^46–48^. In the setting of ACT, IL-7 as a T cell growth factor has been used in *ex vivo* cell culture to expand tumor-reactive T cells^49^. We and others demonstrated that tumor-reactive CD4+ T cells expanded *in vitro* in the presence of IL-7 exhibit superior antitumor potency upon adoptive transfer^50,51^. Besides its role as a T-cell growth factor, the adjuvant effects of IL-7 have recently been recognized. It has been shown that adjuvant IL-7 can enhance the efficacy of cancer vaccine-induced T cell responses by antagonizing multiple immunoinhibitory mechanisms^35,36^. Although an early study reported that administration of rhIL-7 following adoptive immunotherapy led to enhanced antitumor effects in a colon carcinoma xenograft model^52^, the use of IL-7 as an adjuvant for ACT has not been extensively pursued. Here we provide direct evidence that administration of recombinant IL-7 can potentiate the expansion, maintenance and function of adoptively transferred tumor-reactive CD4+ T cells (Fig. 4). We show that both polyfunctional CD4+ effector cells, which arose *in vivo* from infused naïve T cells, and *in vitro*-generated Th1 cells, expressed IL-7Rα (Figs 1B and 5B) and responded to exogenous IL-7 (Figs 4 and 5), while donor CD4+ T cells tolerized in unconditioned tumor-bearing mice failed to regain IL-7Rα expression (Fig. 1A) and were unresponsive to rhIL-7 (Fig. 4A). Our data imply that it is possible to exploit the adjuvant effects of rhIL-7 in human ACT trials if the donor T cells are prepared under conditions that promote their IL-7Rα expression. Alternatively, tumor-specific donor T cells can be engineered to overexpress IL-7Rα to allow them respond to exogenous IL-7 after transfusion. In line with this, a recent study reported that IL-7 selectively favors the expansion, persistence and effector function of T cells co-expressing a GD2-specific chimeric antigen receptor (CAR) and IL-7Rα^53^.

In summary, we present evidence that CTX-based non-myeloablative preparative chemotherapy does not lead to significant increase in IL-7 availability in mice. However, CTX chemotherapy creates an environment that drives robust effector differentiation of adoptively transferred tumor-specific CD4+ T cells, giving rise to polyfunctional CD4 effector cells capable of responding to exogenous IL-7. Our study indicates that although the generation of polyfunctional CD4+ effector cells does not rely on a surge of host-derived IL-7, the magnitude of clonal expansion and long-term survival of CD4+ effector cells can be strengthened by supplementing exogenous IL-7. Our findings provide a strong rationale for using rhIL-7 as an adjuvant to augment the efficacy of CD4+ T-cell adoptive immunotherapy.

## Methods

### Mice

BALB/c mice (Thy1.2+/+) of 4 to 6 weeks of age were purchased from Charles River. 6.5 TCR-Tg mice on a BALB/c (Thy1.1+/+) background expressing an αβTCR specific for amino acids 110–120 from influenza hemagglutinin (HA) presented by IE^d^ were described previously^26^. DO11.10 mice and CD45.1 mice on the BALB/c background were purchased from the Jackson Laboratory. Rag2KO mice on the BALB/c background were purchased from Taconic. All animal experiments were approved by the Institutional Animal Care and Use Committee (IACUC) of Augusta University and performed in accordance with the approved guidelines and regulations.

### Antibodies and flow cytometry analysis

The following fluorochrome-conjugated antibodies were used for flow cytometry: anti-mouse PD1-PE (RMP1–30), IL-7Rα-APC (A7R34), CD4-APC/Cy7 (RM4–5), CD8-PE/Cy7 (53.6.7), CD45.1-PE (A20), CD40L (MR1), IFNγ-APC (XMG1.2), IFNγ-FITC (XMG1.2), TNFα-PE (MP6-XT22), TNFα-PE/Cy7 (MP6-XT22), IL-2-PE (JES6–5H4), and control IgG mAbs were purchased from Biolegend. Thy1.1-perCP (OX-7) were purchased from BD. Foxp3-APC staining kit was purchased from eBiosciences. CellTrace^TM^ CFSE cell proliferation kit was purchased from Invitrogen. Recombinant human interleukin 7 (rhIL-7, CYT107) was provided by Cytheris. Recombinant mouse IL-2 (rmIL-2) and IL-7 (rmIL-7) were purchased from Biolegend. Anti-mouse IL-2 mAbs (Clone S4B6-1 and JES6-1A12) were purchased from Bio X cell. Single cell suspensions prepared spleen and blood were subjected to flow cytometry analysis as described previously^51^. Flow cytometry data were acquired on a LSRII (BD Biosciences) and analyzed with Flowjo software (Treestar inc.) or BD FACSDiva software (BD Biosciences).

### *In vitro* Th1 CD4+ T cells culture

Spleen cells from 6.5 TCR-Tg mice were stimulated with 2 μg/ml HA peptide using a Th1 polarizing cytokine cocktail which contained 10 ng/ml hIL12, 10 ng/ml IFNγ and 20 U/ml hIL2. 7 days later, cells were harvested and dead cells were removed by centrifugation using Ficoll-Paque density gradient medium (GE Healthcare Life Sciences, Uppsala, Sweden). The viable cells were recovered and restimulated with peptide-pulsed fresh splenocytes in the presence of the Th1 polarizing cytokine cocktail. After an additional 3-day culture, viable T cells were enriched by Ficoll and used for adoptive transfer.

### Tumor cells and animal tumor models

The generation and maintenance of HA-expressing A20 tumor cell line (A20HA) was described previously^25^. To establish systemic tumor in mice, A20HA tumor cells were injected to BALB/c mice (1 × 10^6^ per mouse) via tail vein. For adoptive T-cell transfer, spleens and lymph nodes from HA-TCR Tg mice were harvested to enrich for CD4+ T cells by MACS (Miltenyi Biotec). A total of 2.5 to 3 × 10^6^ TCR Tg CD4+ T cells were injected intravenously into each recipient. For Th1 cell adoptive transfer experiments, the subcutaneous A20HA tumor model was used for the convenience of tumor growth monitoring. Specifically, 1 × 10^6^ A20HA tumor cells were subcutaneously implanted to the flank of mice. Mice received the indicated treatments when tumor sizes reached ~170 mm^2^ (length × width). Cyclophosphamide (Sigma) was dissolved in PBS and intraperitoneally injected to mice at the dose of 150 mg/kg unless otherwise specified. rhIL-7 was subcutaneously injected to mice at 10 ug/injection. IL-2/anti-IL-2 mAb complexes (IL-2C) were prepared by incubating 1 ug recombinant mouse IL-2 and 5 ug anti-mouse IL-2 mAb (clone S4B6-1) for 30 minutes at 37 °C, and administrated intraperitoneally in a final volume of 200 ul. *In vivo* neutralization of IL-2 was conducted by i.p. injection of 200 ug anti-mouse IL-2 mAb (clone S4B6-1) and 200 ug anti-mouse IL-2 mAb (clone JES6-1A12) in 200 ul PBS. Isotype control was injected for comparison.

### Quantification of mouse serum IL-7

Serum was collected via the retro-orbital vein from anesthetized mice. Serum IL-7 concentration was measured by a IL-7-dependent cell line, 2E8 (ATCC) as previously described^4^. Briefly, 2E8 cells were labeled with 0.5 uM CFSE and seeded in a round-bottom 96-well plate (10^5^ cells/well in 200 ul medium) in the presence of serum samples, and serially diluted rmIL-7 as the standards. After 3 days in culture, cell proliferation was measured by CFSE dilution using flow cytometry. The standard curve is generated by plotting the percent of divided 2E8 cells against the concentration of rmIL-7. The concentrations of IL-7 in serum samples are calculated based on the regression equation derived from the standard curve.

### Statistical Analysis

Data were analyzed using Prism 4.0 (GraphPad Software, Inc.). The statistical significance of the results was determined using the Student’s t test. Differences in mouse survival among different treatment groups were analyzed using the Gehan-Breslow-Wilcoxon test. P values less than 0.05 were considered statistically significant.
